# GR-MAPPO Algorithm for Perimeter Defense Problem in Multi-Agent Systems

**DOI:** 10.3390/e28060659

**Published:** 2026-06-09

**Authors:** Huihui Tan, Shuang Zhang, Shiwei Lin, Bomin Huang

**Affiliations:** College of Computer Engineering, Jimei University, Xiamen 361021, China

**Keywords:** multi-agent system, perimeter defense, deep learning, reinforcement learning

## Abstract

Multi-agent perimeter defense plays a critical role in cooperative defense scenarios in unmanned swarms. However, existing deep reinforcement learning approaches struggle to effectively exploit both coordination and temporal information under constrained local communication, and they lack generalization capability under dynamic variations in swarm size. To address these challenges, this paper proposes a multi-agent reinforcement learning strategy that integrates coordination under local communication constraints with spatiotemporal feature modeling. Specifically, a GraphSAGE-based spatial aggregation module is employed to enhance information exchange among defenders, while a GRU-based temporal encoding module processes historical observation sequences to improve coordination and anticipatory capability. Furthermore, to overcome scalability limitations, the inductive node-level aggregation mechanism enables agents to adapt to varying numbers of local neighbors, eliminating dependence on a fixed swarm size. Experimental results demonstrate that the proposed GR-MAPPO consistently improves capture performance under limited communication and exhibits better performance retention under cross-scale transfer across different swarm scales.

## 1. Introduction

Recently, the perimeter defense problem, as a typical game problem in multi-agent systems, has received significant attention. This problem is widely applied in scenarios such as maritime surveillance, drone air combat, and military reconnaissance [1,2,3]. Current research on the multi-agent perimeter defense problem is primarily based on analytical methods. Shishika et al. [4] proposed a multi-defender perimeter defense framework based on cooperative geometric analysis and differential games, addressing the limitations of a single defender in countering highly maneuverable attackers. Subsequently, in 2017, Chen et al. [5] introduced a pairwise decomposition approach for multi-agent reach–avoid games, which decomposes a multi-agent game into a set of two-player subgames, thereby mitigating role overlap and improving resource utilization among defenders. In a related study, Yan et al. [6] developed a cooperative capture strategy for a two-defender–one-attacker scenario using analytically constructed barrier functions, enabling defenders to form coordinated pincer maneuvers and transitioning perimeter defense from single-point interception to cooperative containment. When extending the problem to multi-defender versus multi-attacker scenarios, research has increasingly focused on task allocation and hierarchical coordination. Pierson [7] proposed a target assignment-based perimeter defense method to address the ambiguity in defender–attacker matching. In 2022, Yan et al. [8] further proposed a matching decomposition-based capture strategy for multi-agent reach–avoid games, decoupling high-level assignment from low-level trajectory optimization, thereby reducing computational complexity. Von Moll et al. [9] investigated multi-pursuer perimeter defense differential games by leveraging dominance region analysis and analytical geometry, deriving optimal interception strategies and explicitly characterizing defender dominance regions as well as analytical conditions for successful attacker penetration. This work extends classical single-pursuer perimeter defense theory to cooperative multi-pursuer settings.

Although these analytical and differential game-based methods can achieve theoretically optimal solutions in structured environments, they typically rely on precise modeling and full state information, and suffer from rapidly increasing computational complexity as the number of agents grows. This limits their applicability in high-dimensional and dynamic adversarial scenarios [10]. Consequently, recent research has increasingly shifted toward deep reinforcement learning approaches to enhance adaptability in complex environments.

In perimeter defense scenarios, agents typically have access only to local observations, and their communication capabilities are often limited or even absent. Under such conditions, policies without explicit coordination mechanisms tend to suffer from redundant interceptions or missed targets, thereby degrading overall cooperative performance. To address the need for information exchange under constrained communication, Jiang et al. [11] introduced graph neural networks (GNNs) [12] into multi-agent perimeter defense to capture inter-agent topological relationships and enhance cooperative representation. Niu et al. [13] further proposed the MAGIC framework, which leverages graph attention mechanisms to dynamically adapt communication topologies [14]. To further address complex information interactions and model scalability, Li et al. [15] proposed a hierarchical graph attention actor-critic method that maintains a constant embedding dimensionality irrespective of the number of agents, thereby enhancing adaptability and transferability across varying swarm scales. Meanwhile, perimeter defense tasks are inherently partially observable, requiring agents to utilize historical information for effective decision-making. Recurrent neural networks (RNNs) [16], through recursive hidden-state propagation, offer an effective mechanism for modeling temporal dependencies [17]. Jiang et al. [18] proposed an LSTM-MAPPO algorithm for UAV cooperative air combat maneuvering, incorporating long short-term memory (LSTM) units into the Actor–Critic architecture to enhance historical information extraction and decision performance.

However, existing studies largely treat spatial coordination features and temporal evolution features in isolation. Recent work [19] highlights that purely temporal models (e.g., RNN/LSTM) can only encode individual agents’ historical trajectories and fail to capture dynamic topological relationships in multi-agent systems, whereas graph-based spatial methods typically rely on single-step observations and lack the ability to model long-term temporal behaviors. This decoupled approach to spatiotemporal feature extraction limits the ability of current approaches to simultaneously capture long-term attacker maneuvering patterns and the dynamic cooperative structure of defender swarms. In practical unmanned swarm confrontation tasks, the number of agents may vary due to attrition, communication constraints, or dynamic reinforcements, resulting in inherently dynamic system scales [20]. Nonetheless, constrained by underlying network architectures, existing multi-agent defense strategies generally suffer from significant scalability limitations. Traditional multi-agent reinforcement learning algorithms based on multilayer perceptrons (MLPs), such as standard MAPPO [21] and MADDPG [22], rely on centralized value networks with fixed-dimensional global joint state inputs. When MLPs are used to encode inter-agent interactions, they also require a fixed number of neighbors and input dimensions. Recent studies on zero-shot scalability in swarm intelligence, including the systematic analysis by Aguzzi et al. [23] and the cross-environment zero-shot cooperation framework proposed by Jha et al. [24], have explicitly identified this limitation. When the number of agents changes, the dimensionality of global state representations and interaction modeling varies accordingly, breaking the consistency of policy network inputs and severely restricting cross-scale zero-shot generalization in dynamic and open adversarial environments.

In light of these challenges, enabling effective spatiotemporal coordination under local communication constraints while maintaining cross-scale generalization has become a key challenge in multi-agent perimeter defense. In summary, deep reinforcement learning has emerged as a promising paradigm for perimeter defense. Compared with analytical methods, several limitations remain: (1) under local communication constraints, cooperative and temporal information is still not fully exploited and (2) existing coordination strategies heavily rely on a fixed number of agents and lack flexibility and generalization when facing dynamically changing swarm scales. To address these issues, the main contributions of this paper are summarized as follows:

1. A spatiotemporal cooperative perimeter defense strategy, termed GR-MAPPO, is proposed. To tackle inadequate exploitation of cooperative information among defenders and inadequate modeling of attacker behavior evolution under limited communication, a GraphSAGE-based spatial aggregation module and a GRU-based temporal encoding module are incorporated into the MAPPO framework. This design enhances cooperative decision-making, improves the anticipatory modeling of attacker behavior, resulting in improved interception performance.
2. A multi-agent cooperative decision-making framework with cross-scale zero-shot generalization capability is developed. By leveraging the inductive graph learning property of GraphSAGE, agents can adapt to varying numbers of local communication neighbors. This alleviates the structural dependence of traditional MLP-based methods on fixed-dimensional inputs, enabling zero-shot transfer across different swarm scales while maintaining stable cooperative interception performance.

## 2. Problem Formulation

### 2.1. Environment Setup

**Figure 1:**
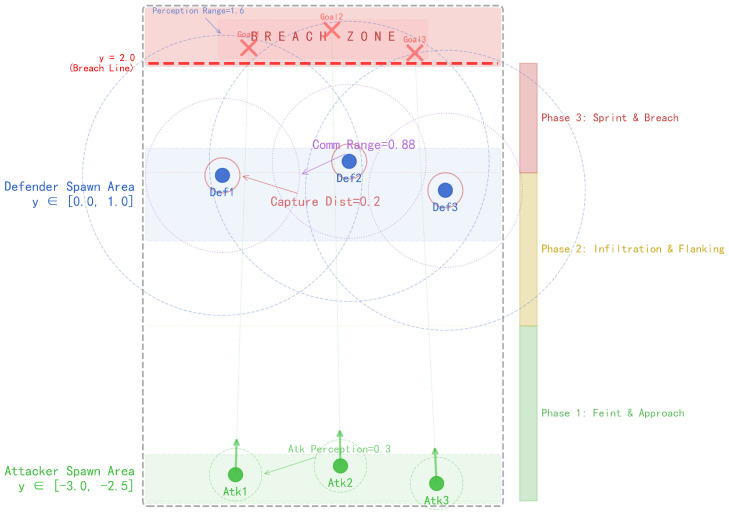
Illustration of the perimeter defense scenario.

As illustrated in Figure 1, a bounded two-dimensional plane is considered as the adversarial environment. To maintain a consistent interaction density across different agent scales, an adaptive width scaling mechanism is adopted. Using three defenders as the baseline configuration with environment width $W_{\mathrm{base}}$, the horizontal space allocated per defender is fixed at $W_{\mathrm{base}}/3$. When the number of defenders is *N*, the total environment width is adaptively adjusted as:

(1) $$W_{\mathrm{env}}=N\times W_{\mathrm{base}}/3$$

The range of the X-axis is defined as $\left[-W_{\mathrm{env}}/2,W_{\mathrm{env}}/2\right]$, while the Y-axis range remains invariant with respect to the number of agents. A horizontal boundary line is located at $y=y_{\mathrm{line}}$ in the upper region of the environment.

Within the environment, there exists a set of defenders $D=\{D_{1},D_{2},\ldots ,D_{N}\}$ and a set of attackers $I=\{I_{1},I_{2},\ldots ,I_{M}\}$. All agents share the same radius $r_{\mathrm{agent}}$. Attackers are initialized in the lower region (y∈[−3.0,−2.5]) and aim to reach and cross the boundary line, whereas defenders are initialized in the central region (y∈[0,1.0]) and aim to intercept attackers before they cross the boundary. This adaptive scaling mechanism ensures consistent defender spacing and attacker passability across different scales (e.g., 3v3, 10v10, and 20v20).

### 2.2. Agent Dynamics

In this adversarial environment, defender *i* follows an acceleration-controlled kinematic model:

(2) $$\begin{array}{cc} v_{i}^{D}\left(t+1\right) & \;=\;v_{i}^{D}\left(t\right)+a_{i}\left(t\right)\cdot \Delta t\\ p_{i}^{D}\left(t+1\right) & \;=\;p_{i}^{D}\left(t\right)+v_{i}^{D}\left(t+1\right)\cdot \Delta t \end{array}$$

where $p_{i}^{D}\left(t\right)$ and $v_{i}^{D}\left(t\right)$ denote the position and velocity of defender *i* at time step *t*, respectively. The control input $a_{i}\left(t\right)$ is generated by the reinforcement learning policy, and Δt is the simulation time step. The velocity is constrained by a maximum speed $v_{\max}^{D}$.

For attacker *j*, the kinematic model is defined as:

(3) $$p_{j}^{I}\left(t+1\right)=p_{j}^{I}\left(t\right)+v_{j}^{I}\left(t\right)\cdot \Delta t$$

where $p_{j}^{I}\left(t\right)$ denotes the position of attacker *j*, and $v_{j}^{I}\left(t\right)$ is determined by a predefined rule-based policy, with its magnitude bounded by $v_{\max}^{I}$.

### 2.3. Environment Interaction and Termination Conditions

The state transition is governed by explicit interaction rules and physical constraints. When the Euclidean distance between any defender *i* and any active attacker *j* is smaller than the capture radius $R_{\mathrm{cap}}=0.2$, i.e.,

(4) $$∥p_{i}^{D}-p_{j}^{I}∥<R_{\mathrm{cap}}$$

the attacker *j* is considered successfully intercepted, and both agents are removed from the environment.

If any uncaptured attacker crosses the boundary line, i.e.,

(5) $$p_{jy}\geq y_{\mathrm{line}}=2.0$$

it is considered a successful breach and removed from the environment, triggering a penalty for the defenders.

The X-axis boundary is $\left[-W_{\mathrm{env}}/2,W_{\mathrm{env}}/2\right]$. When an agent exceeds the boundary, its position is clipped to remain within valid limits. An episode terminates when either: (1) all attackers are no longer active (captured or have breached the boundary), or (2) the maximum time step $T_{\max}$ is reached.

### 2.4. Multi-Stage Artificial Potential Field Strategy for Attackers

To simulate complex dynamic behaviors, attackers follow a scripted policy based on a multi-stage artificial potential field (APF) method [25]. The behavior is divided into three stages according to the longitudinal progress $\mu_{y}$, simulating a progressive attack process from a probing approach, to gap searching, and finally a full sprint:

(6) $$\mu_{y}=\frac{p_{jy}^{I}-y_{\mathrm{spawn}}}{y_{\mathrm{line}}-y_{\mathrm{spawn}}}$$

where $p_{jy}^{I}$ is the current longitudinal coordinate of the attacker, $y_{\mathrm{spawn}}=-2.0$ is the starting line, and $y_{\mathrm{line}}=2.0$ is the boundary line. The value of $\mu_{y}$ is clipped to [0,1], with larger values indicating proximity to the boundary. Based on $\mu_{y}$, the stages are defined as follows:

**Stage 1** ($\mu_{y}<\mu_{th1}$): Attackers approach the boundary at a low speed while exerting large lateral oscillations to create deceptive maneuvers, misleading defenders about their true breakthrough direction. The stage modulation coefficients reduce the attractive component while amplifying the repulsive and lateral deception components, thereby encouraging evasive and deceptive maneuvers during the early probing phase.

**Stage 2** ($\mu_{th1}\leq \mu_{y}<\mu_{th2}$): The stage modulation coefficients rebalance the attractive, lateral deception, and ally interaction components to support coordinated gap-searching behaviors during the intermediate attack phase. A gap-searching mechanism is introduced, where attackers search for breakthrough gaps by detecting the opposite deviation of the average lateral position of nearby defenders. Furthermore, the ally interaction force is amplified by a factor of 2 during Stage 2, strengthening local coordination among nearby attackers. Specifically, attackers are attracted toward neighboring allies within an intermediate distance range to maintain cooperative group movement, while short-range repulsion is simultaneously applied to avoid excessive clustering and collision. Combined with the gap-searching mechanism and stage-dependent lateral oscillation, this design encourages multiple attackers to perform simultaneous breakthroughs from different lateral directions, thereby dispersing defensive attention and increasing defensive decision difficulty.

**Stage 3** ($\mu_{y}\geq \mu_{th2}$): The stage modulation coefficients strengthen the goal-attraction component while relatively suppressing repulsive and lateral perturbation effects, encouraging direct sprinting behavior toward the boundary. Attackers sprint toward the boundary while maintaining slight lateral perturbation. If the threat level from nearby defenders exceeds $\lambda_{\mathrm{threat}}^{th}$ and $\mu_{y}>\mu_{\mathrm{sprint}}$, attackers will bypass the distance condition and directly switch to Stage 3 for a full sprint.

All stages share a base force field model comprising the following components. The attractive force $F_{\mathrm{att}}$ pulls the attacker toward its goal $p_{\mathrm{goal}}$:

(7) $$F_{\mathrm{att}}=-\zeta \left(p_{j}^{I}-p_{\mathrm{goal}}\right)$$

where ζ is the attractive gain coefficient, and $p_{j}^{I}$ is the current position of attacker *j*. The repulsive force $F_{\mathrm{rep}}$ drives the attacker away from defenders within its sensing range:

(8) $$F_{\mathrm{rep}}=\sum_{j\in N_{I}}\frac{\eta_{\mathrm{rep}}}{d_{ij}^{2}}\left(\frac{1}{d_{ij}}-\frac{1}{d_{0}}\right){\hat{e}}_{j}$$

where $\eta_{\mathrm{rep}}$ is the repulsive gain coefficient, $d_{ij}$ is the distance between attacker *i* and defender *j*, $d_{0}$ is the attacker’s sensing radius, ${\hat{e}}_{j}$ is the unit vector pointing away from the defender, and $N_{I}$ is the set of defenders within sensing range.

The lateral deception force $F_{\mathrm{lat}}$ generates unpredictable lateral movements based on a time-varying sinusoidal oscillation:

(9) $$F_{\mathrm{lat}}=\lambda_{\mathrm{threat}}\cdot A\left(\varphi \right)\cdot \mathrm{sgn}\left[\sin(\omega_{\varphi }\cdot t+\psi^{\mathrm{phase}})\right]\cdot {\hat{e}}_{x}$$

where A(φ) and $\omega_{\varphi }$ are the amplitude and frequency determined by the current stage φ; and $\psi^{\mathrm{phase}}$ is a random phase shift based on the attacker’s ID to ensure diverse motion patterns. $\lambda_{\mathrm{threat}}\in \left[0,1\right]$ is the threat level calculated based on the distance to the nearest defender; Specifically, the threat level is defined as:

(10) $$\lambda_{\mathrm{threat}}=\max\left(0,1-\frac{d_{\min}}{R_{\det}}\right)$$

where $d_{\min}$ denotes the distance between the attacker and the nearest defender within the sensing range.

Additionally, an ally interaction force $F_{\mathrm{ally}}$ exists among attackers. It generates an attractive force to promote cooperative breakthrough when the inter-ally distance is within $\left(d_{\mathrm{ally}}^{\min},d_{\mathrm{ally}}^{\max}\right)$, and a repulsive force to avoid collision when the distance is less than $d_{\mathrm{ally}}^{\min}$. The interaction force is defined as:

(11) $$F_{\mathrm{ally}}=\left\{\begin{array}{cc} \eta_{\mathrm{att}}{\hat{e}}_{ij}, & d_{\mathrm{ally}}^{\min}<d_{ij}<d_{\mathrm{ally}}^{\max}\\ {-\eta }_{\text{rep\_ally}}{\hat{e}}_{ij}, & d_{ij}\leq d_{\mathrm{ally}}^{\min}\\ 0, & \mathrm{otherwise} \end{array}\right.$$

where $d_{ij}$ denotes the inter-attacker distance, ${\hat{e}}_{ij}$ is the unit vector pointing from attacker *i* to attacker *j*, and $\eta_{\mathrm{att}}$ and $\eta_{\text{rep\_ally}}$ are the ally attraction and ally repulsion coefficients, respectively. The attractive component encourages nearby attackers to maintain coordinated movement and perform cooperative breakthroughs, while the repulsive component prevents excessive clustering and collision.

As a result, attackers tend to maintain loose local formations and initiate coordinated breakthroughs from multiple lateral directions during Stage 2, thereby dispersing defensive attention and increasing the difficulty of cooperative defense.

Finally, the velocity components are modulated by stage-specific coefficients and superimposed with Gaussian noise $o∼N(0,\sigma_{o}^{2})$ to generate the synthetic velocity command:

(12) $$v_{j}\left(t\right)=f_{\mathrm{norm}}\left(\alpha_{\varphi }F_{\mathrm{att}},\beta_{\varphi }F_{\mathrm{rep}},\gamma_{\varphi }F_{\mathrm{lat}},\delta_{\varphi }F_{\mathrm{ally}}\right)+o$$

where $\alpha_{\varphi },\beta_{\varphi },\gamma_{\varphi },\delta_{\varphi }$ are the corresponding field coefficients for each stage φ∈{1,2,3}, and $f_{\mathrm{norm}}\left(\cdot \right)$ is a clipping function that bounds the synthetic velocity within the maximum speed $v_{\max}^{I}$.

### 2.5. Optimization Objective

To quantitatively evaluate defense performance, the Mean Interception Rate (MIR) is defined as the ratio of successfully captured attackers at the end of an episode, $M_{cap}$, to the initial total number of attackers, *M*:

(13) $$\mathrm{MIR}=\frac{M_{cap}}{M}$$

Given the environmental constraints and dynamic attacker strategies described above, the objective of this paper is to develop a deep reinforcement learning-based cooperative interception policy for the defenders. Under local observation, the proposed policy is designed to maximize the interception rate within the time horizon $T_{\max}$.

## 3. Algorithm Framework

**Figure 2:**
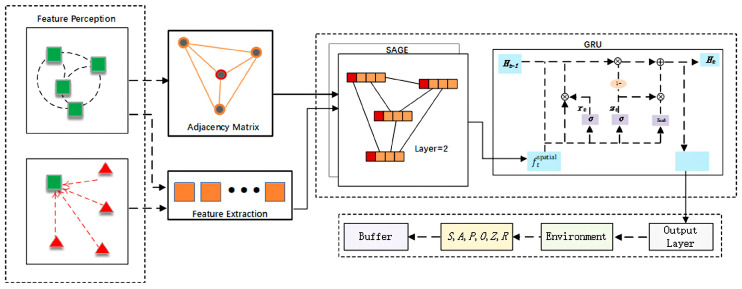
Architecture of the GR-MAPPO algorithm.

This section details the proposed GR-MAPPO boundary defense algorithm under local communication conditions. The algorithm is based on the MAPPO training framework. Its core design lies in constructing the topological relationship among defenders via a Graph Neural Network (GNN) to achieve the structured aggregation of friendly features, and utilizing a Gated Recurrent Unit (GRU) to perform temporal modeling on historical observation sequences to capture the movement trends of attackers. The overall architecture of the algorithm is illustrated in Figure 2.

### 3.1. State and Action Space

Since each defending agent can only access local information within its sensor range and cannot directly access the complete environmental state, this paper models the multi-agent cooperative interception problem as a Partially Observable Markov Decision Process (POMDP), formally defined as a 7-tuple $\langle S,\left\{A_{i}\right\},P,\left\{O_{i}\right\},Z,R,\gamma \rangle$. Here, *S* is the global state space containing complete information such as the true positions and velocities of all agents; $A_{i}$ is the action space of agent *i*; $P:S\times A_{1}\times \cdots \times A_{N}\to \Delta \left(S\right)$ is the state transition function; $O_{i}$ is the observation space of agent *i*; $Z:S\times N\to O_{i}$ is the observation function, which defines the local observation $o_{i}=Z\left(s,i\right)$ obtained by agent *i* under the global state *s*; *R* is the shared reward function, detailed in Section 3.4; and γ is the discount factor. Specifically, the observation function *Z* restricts defending agent *i* to only observe the information of other agents within its sensing radius $R_{\det}$. Based on the above POMDP model, this section specifies the local observation vector and action space required by the algorithm.

In terms of information interaction design, it is assumed that there is no predefined explicit communication protocol among defending agents; instead, information interaction is implicitly achieved via the GNN within the communication range. Each defender *i* can only obtain the positions and velocities of attackers within its sensing radius $R_{\det}$, and implicitly acquire the positions and velocities of friendly defenders within its communication radius $R_{\mathrm{com}}$. That is, for any defender *i*, the states of other agents located outside its sensing and communication ranges are unobservable. This paper designs the defender’s observation information as a concatenation of its own state and enemy features, while the fusion of friendly collaborative information is implicitly handled by the GNN through a message-passing mechanism. Specifically, for the *i*-th defending agent, assume it can detect a maximum of $k_{a}$ attackers within its sensing range $R_{\det}$, satisfying the constraint $k_{a}=\min\left(M,3\right)$. The local observation vector $o_{i}$ is constructed as follows:

(14) $$o_{i}=\left[\left(v_{ix}^{D},v_{iy}^{D}\right),\left(p_{ix}^{D},p_{iy}^{D}\right),\left(e_{i,1},\ldots ,e_{i,k_{a}}\right)\right]$$

The self-velocity $\left(v_{ix}^{D},v_{iy}^{D}\right)$ and self-position $\left(p_{ix}^{D},p_{iy}^{D}\right)$ constitute 4 dimensions. Each enemy feature slot $e_{i,j}$ is a 5-dimensional vector:

(15) $$e_{i,j}=\left[{\mathrm{valid}}_{j},\left(\Delta p_{x},\Delta p_{y}\right),\left(v_{jx}^{I},v_{jy}^{I}\right)\right]$$

where ${\mathrm{valid}}_{j}\in \left\{0,1\right\}$ is a validity flag, set to 1 when the *k*-th nearest attacker exists within the sensing range, and 0 otherwise, with all remaining components padded with zeros. $\left(\Delta p_{x},\Delta p_{y}\right)$ represents the positional difference of attacker *j* relative to defender *i*, and $\left(v_{jx}^{I},v_{jy}^{I}\right)$ is the velocity of the attacker. Attackers are sorted by their Euclidean distance to defender *i* from nearest to farthest before being filled into the slots.

Therefore, the total dimension of the observation vector is $d_{o}=4+5k_{a}$. The above observation does not include the state information of friendly defenders. Friendly information will be fused through the mechanism described in Section 3.2. This processing approach separates the heterogeneous information of adversaries and allies into different channels, avoiding the feature confusion caused by simple concatenation.In addition to the observation design, the action space of each agent is defined as follows.

For action representation, the continuous acceleration space is discretized into a set of five basic strategic actions $U=\{u_{0},u_{1},u_{2},u_{3},u_{4}\}$, corresponding to unit accelerations in five directions: stationary, left, right, down, and up, respectively:

(16) $$u_{0}=\left(0,0\right),u_{1}=\left(-1,0\right),u_{2}=\left(1,0\right),u_{3}=\left(0,-1\right),u_{4}=\left(0,1\right)$$

The actual acceleration is scaled by a sensitivity coefficient κ as $a_{i}=\kappa \cdot u_{k}$. The velocity and position are updated according to kinematic equations and are bounded by a maximum velocity constraint $v_{\max}^{D}=0.5\;m/s$.

### 3.2. Spatial Feature Aggregation Based on GraphSAGE

The GNN needs to dynamically construct a communication graph reflecting the spatial topological relationship of the defenders at each decision time step. Based on the communication range $R_{\mathrm{com}}$ of the defenders, this paper constructs an undirected communication graph $G=(V_{t},E_{t})$. The node set $V_{t}$ consists of all surviving defenders at the current time step, i.e., $V_{t}=\left\{D_{i}|D_{i}\;\mathrm{is}\;\mathrm{active}\right\}$. The initial feature of each node $D_{i}$ is its local observation vector $o_{i}$. The edge set $E_{t}$ is determined by the Euclidean distance between defenders. When the distance between two defenders $D_{i}$ and $D_{j}$ satisfies $∥p_{i}^{D}-p_{j}^{D}∥\leq R_{\mathrm{com}}$, bidirectional edges $\left(D_{i},D_{j}\right)\in E_{t}$ and $\left(D_{j},D_{i}\right)\in E_{t}$ are established between them. The communication range is set to $R_{\mathrm{com}}=0.22W_{\mathrm{env}}$, where $W_{\mathrm{env}}$ is the environmental width of the current scenario.

After constructing the communication topology graph, this paper employs GraphSAGE as the specific implementation of the GNN to perform structured aggregation of collaborative features among defenders. The designed GNN module consists of two GraphSAGE convolutional layers, each equipped with residual connections and Layer Normalization (LayerNorm). Let the feature representation of node $D_{i}$ at the *l*-th layer be $h_{i}^{\left(l\right)}$, where $h_{i}^{\left(0\right)}=o_{i}$ is the raw observation vector after LayerNorm. The message passing and update process of the *l*-th GraphSAGE layer is as follows: first, the neighbor features are mean-aggregated:

(17) $$h_{N(i)}^{\left(l\right)}=\mathrm{MEAN}\left(\left\{h_{j}^{\left(l-1\right)},\forall j\in N\left(i\right)\right\}\right)$$

where N(i) is the neighbor set of node $D_{i}$ in graph $G_{t}$. Subsequently, the aggregated neighbor features are concatenated with the node’s own features, and the updated node representation is obtained through linear transformation and non-linear activation:

(18) $$h_{i}^{\left(l\right)}=\tanh\left(W^{\left(l\right)}\cdot \mathrm{CONCAT}\left(h_{i}^{\left(l-1\right)},h_{N(i)}^{\left(l\right)}\right)\right)$$

where $W^{\left(l\right)}$ is the learnable weight matrix of the *l*-th layer, and tanh(·) is the Tanh activation function. To stabilize the GNN training process and prevent feature degradation, residual connections and LayerNorm are introduced after each GraphSAGE convolution layer. Specifically, the complete computation process of the first layer is:

(19) $$\begin{array}{cc} {\tilde{h}}_{i}^{\left(1\right)} & \;=\mathrm{SAGEConv}(G_{t},h_{i}^{\left(0\right)})\\ h_{i}^{\left(1\right)} & \;=\tanh\left(\mathrm{LayerNorm}\left({\tilde{h}}_{i}^{\left(1\right)}\right)\right)+W_{\mathrm{res}}h_{i}^{\left(0\right)} \end{array}$$

When the input dimension $d_{o}$ differs from the hidden dimension $d_{h}$, the residual connection aligns the dimensions via a linear projection $W_{\mathrm{res}}$; when they are identical, an identity mapping is applied directly. The computation process of the second layer is similar, with input $h_{i}^{\left(1\right)}$ and output $h_{i}^{\left(2\right)}$. After two GraphSAGE layers, the feature $h_{i}^{\left(2\right)}$ of each defender node encodes its own local observation and integrates the state information of friendly defenders within a two-hop communication neighborhood.

### 3.3. Temporal Modeling Based on GRU

As described in Section 2, the attacker’s strategy is divided into three stages according to $\mu_{y}$. To model the temporal information during the behavioral evolution process, a GRU is introduced after the GraphSAGE spatial feature extraction module to perform temporal encoding on the spatially aggregated feature sequence. Specifically, the GRU module takes the GraphSAGE output spatial aggregation feature $f_{i}^{\mathrm{spatial}}\in R^{d_{h}}$ and the previous hidden state $h_{i,t-1}\in R^{d_{h}}$ as inputs, and outputs the current hidden state $h_{i,t}\in R^{d_{h}}$. This paper uses $h_{i,t}$ as the temporally enhanced feature $f_{i}^{\mathrm{temporal}}\in R^{d_{h}}$, i.e., $f_{i}^{\mathrm{temporal}}=h_{i,t}$. A single-layer GRU is utilized, where its output at a single time step is exactly the updated hidden state. In the subsequent policy network, $f_{i}^{\mathrm{temporal}}$ is fed into the action output layer, while $h_{i,t}$ is passed as the hidden state to the next time step. During training, to prevent the hidden state from crossing episode boundaries, a masking mechanism is introduced before the hidden state update:

(20) $$h_{i,t-1}^{\prime }=h_{i,t-1}⊙{\mathrm{mask}}_{t}.$$

When ${\mathrm{mask}}_{t}=0$, the hidden state is cleared; when ${\mathrm{mask}}_{t}=1$, the hidden state propagates normally.

### 3.4. Reward Function

The design of the reward function directly affects the training efficacy and final performance of the reinforcement learning algorithm. To alleviate the training difficulties caused by sparse rewards, this paper designs a composite reward mechanism consisting of a distance-guided term, a breach penalty term and a capture reward term. The total reward $r_{i,t}$ for defending agent *i* at time step *t* consists of the following components:

(1) Distance-guided reward. To encourage defenders to actively approach attackers, a dense penalty proportional to the distance to the nearest active attacker within the sensing range is applied:
  (21) $$r_{i,dist}=-\min_{j\in M_{i}}∥p_{i}^{D}-p_{j}^{I}∥\cdot \frac{W_{base}}{W_{env}}$$
  where $M_{i}$ is the set of active attackers within the sensing range of agent *i*, and $W_{base}/W_{env}$ is the scenario normalization coefficient to ensure consistency of reward magnitudes across different scenario scales. When there are no active attackers in the field, this term is zero.
(2) Breach penalty. When an attacker successfully crosses the defense boundary line, a one-time penalty is given to all defenders:
  (22) $$r_{i,breach}=\left\{\begin{array}{cc} \lambda_{breach} & \mathrm{if}\;\mathrm{attacker}\;j\;\mathrm{successfully}\;\mathrm{breaches}\\ 0 & \mathrm{otherwise} \end{array}\right.$$
  By maintaining a set of penalized instances, it is ensured that each breach event triggers a penalty only once, avoiding duplicate calculations.
(3) Capture reward. When an attacker is successfully captured, the reward consists of two parts: a team reward and an individual incentive:
  (23) $$r_{i,capture}=r_{i,team}+r_{i,individual}$$
  The team reward $r_{i,team}$ is distributed to all defenders, reflecting the collaborative incentive of collective gains; the individual incentive $r_{i,individual}$ is only awarded to the defender that actually executes the capture, encouraging defenders to actively approach and complete interception actions.

In summary, the total reward for defending agent *i* at time step *t* is:

(24) $$r_{i,t}=r_{i,dist}+r_{i,breach}+r_{i,capture}$$

The distance term provides dense feedback at each time step, mitigating the lack of training signals under purely sparse rewards. The capture reward further distinguishes between team and individual gains, thereby alleviating the unclear credit assignment problem in multi-agent scenarios.

### 3.5. Policy and Value Networks

**Figure 3:**
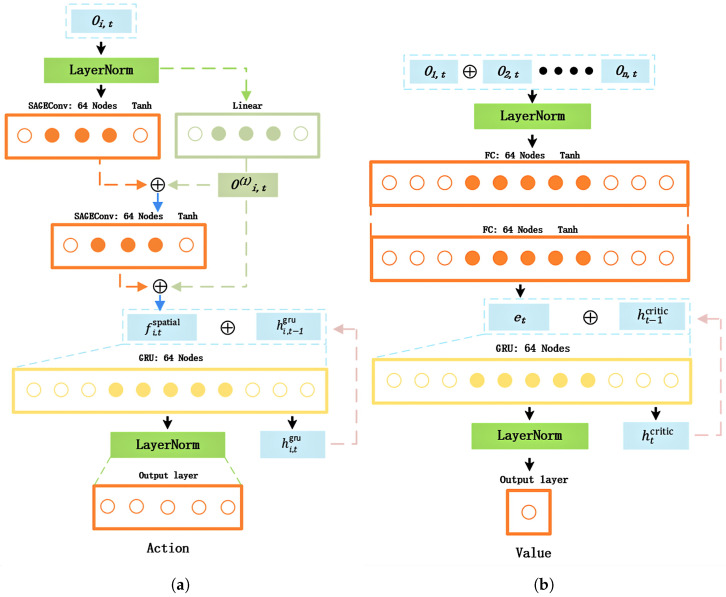
Algorithm architecture. (**a**) Policy network; (**b**) Value network.

The network architecture adopts a Centralized Training with Decentralized Execution (CTDE) framework. Under this framework, the policy and value networks use different information input modalities. For the policy network, its parameters are denoted as ϕ, and all homogeneous defenders share the same set of parameters. As shown in Figure 3a, the policy network consists of three sequentially connected modules:

(1) GraphSAGE Spatial Aggregation Module: Takes the defender’s local observation $o_{i}$ as node features and the communication topology graph *G* as structural input. It aggregates collaborative information from friendly neighbors through two GraphSAGE convolutional layers, outputting a spatial aggregation feature of dimension $d_{h}$. The input observation is first standardized via LayerNorm before entering the temporal modeling module.
(2) GRU Temporal Modeling Module: Receives the output features from GraphSAGE and the hidden state from the previous time step. It fuses historical information via a gating mechanism, outputting temporally enhanced features of dimension $d_{h}$. The GRU output is also processed by LayerNorm.
(3) Action Output Layer (ACTLayer): Maps the features output by the GRU to a probability distribution over the five discrete actions $\pi_{\phi }\left(\cdot |o_{i},G_{t},h_{i,t-1}^{\mathrm{gru}}\right)$. The complete forward computation process of the policy network can be expressed as:
  (25) $$\begin{array}{cc} f_{i}^{\mathrm{spatial}} & \;=\mathrm{GraphSAGE}(G_{t},\mathrm{LayerNorm}\left(o_{i}\right))\\ f_{i}^{\mathrm{temporal}},h_{i,t}^{\mathrm{gru}} & \;=\mathrm{GRU}(f_{i}^{\mathrm{spatial}},h_{i,t-1}^{\mathrm{gru}}⊙{\mathrm{mask}}_{t})\\ \pi_{\phi }\left(\cdot |o_{i},G_{t},h_{i,t-1}^{\mathrm{gru}}\right) & \;=\mathrm{Softmax}\left(W_{\mathrm{act}}\cdot \mathrm{LayerNorm}\left(f_{i}^{\mathrm{temporal}}\right)+b_{\mathrm{act}}\right) \end{array}$$

    For the value network, its parameters are denoted as θ, and all agents share the same set of parameters. As shown in Figure 3b, the value network concatenates the local observations of all defenders into a joint observation ${\tilde{s}}_{t}=\left[o_{1,t},o_{2,t},\ldots ,o_{n,t}\right]$ to approximate the global state as input, reflecting the characteristics of centralized training. For brevity, $s_{t}$ is still used to denote this input in the subsequent formulas. Its structure includes:

(1) MLP Feature Extraction Module: Consists of two fully connected layers (each with $d_{h}$ neurons) and Tanh activation functions, with LayerNorm appended after each layer. The input global state is first standardized via LayerNorm.
(2) GRU Temporal Modeling Module: Identical to the GRU structure in the policy network, used to capture the temporal variation patterns of the global state.
(3) Value Output Layer: Maps the GRU output to a scalar value estimate $V_{\theta }\left(s_{t}\right)$, with the output layer being a single linear neuron. Since the value network can access global state information during the training phase and possesses the capability to model the overall environmental information, it does not need to additionally rely on graph structures for information aggregation.

Parameter Sharing Mechanism: Because all defending agents are homogeneous, this work adopts a parameter sharing strategy—all defending agents share the same policy network parameters ϕ and value network parameters θ. Parameter sharing effectively reduces the number of model parameters and improves the utilization efficiency of training samples. As the number of agents increases, this mechanism also helps improve training efficiency and convergence speed.

It is worth noting that cooperative multi-agent systems relying on information exchange or consensus mechanisms may potentially suffer from communication security vulnerabilities, such as malicious data tampering or adversarial interference. Although the proposed framework assumes a trusted communication environment and mainly focuses on cooperative policy learning efficiency, secure-by-design consensus and robust information exchange mechanisms are important considerations for practical deployment in security-critical perimeter defense scenarios. Recent studies have investigated secure consensus protocols and robustness against structured communication attacks in multi-agent networks [26,27]. Incorporating such secure communication mechanisms into multi-agent perimeter defense frameworks will be considered in future work.

### 3.6. Training Algorithm

This paper uses MAPPO as the foundational training framework, integrating the aforementioned GraphSAGE spatial aggregation and GRU temporal modeling modules. The training process utilizes an on-policy data collection method and Proximal Policy Optimization (PPO) update rules, employing Generalized Advantage Estimation (GAE) to balance bias and variance:

(26) $${\hat{A}}_{i,t}=\sum_{l=0}^{T-t-1}{\left(\gamma \lambda \right)}^{l}\delta_{i,t+l}^{\mathrm{TD}}$$

where $\delta_{i,t+l}^{\mathrm{TD}}=r_{i,t+l}+\gamma V_{\theta }\left(s_{t+l+1}\right)-V_{\theta }\left(s_{t+l}\right)$ is the Temporal Difference (TD) error, γ is the discount factor, and λ∈[0,1] is the GAE smoothing parameter. $V_{\theta }\left(s_{t}\right)$ is the output of the value network, with its full form being $V_{\theta }\left(s_{t},h_{t-1}^{\mathrm{critic}}\right)$. After calculating the advantage function, it must be normalized to improve the stability of the training process. Furthermore, the importance sampling ratio between the new and old policies is defined as:

(27) $$\rho_{i,t}\left(\phi \right)=\frac{\pi_{\phi }\left(a_{i,t}|o_{i},G_{t},h_{i,t-1}^{gru}\right)}{\pi_{\phi_{\mathrm{old}}}\left(a_{i,t}|o_{i},G_{t},h_{i,t-1}^{gru}\right)}$$

The policy network is updated by maximizing the clipped surrogate objective function:

(28) $$L^{\mathrm{CLIP}}\left(\phi \right)=E_{t}\left[\min\left(\rho_{i,t}\left(\phi \right){\hat{A}}_{i,t},\mathrm{clip}\left(\rho_{i,t}\left(\phi \right),1-\epsilon_{\mathrm{clip}},1+\epsilon_{\mathrm{clip}}\right){\hat{A}}_{i,t}\right)\right]$$

where $\epsilon_{\mathrm{clip}}=0.2$ is the clipping hyperparameter. The value network is updated by minimizing the Huber loss, and a clipping mechanism is introduced to prevent drastic jumps in the value function:

(29) $$L^{\mathrm{VF}}\left(\theta \right)=E_{t}\left[\max\left(L_{\delta^{TD}}\left(V_{\theta }\left(s_{t}\right)-{\hat{R}}_{t}\right),L_{\delta_{H}}\left(V_{\theta }^{\mathrm{clip}}\left(s_{t}\right)-{\hat{R}}_{t}\right)\right)\right]$$

where $V_{\theta }^{\mathrm{clip}}=V_{\theta_{\mathrm{old}}}+\mathrm{clip}\left(V_{\theta }-V_{\theta_{\mathrm{old}}},-\epsilon_{\mathrm{clip}},\epsilon_{\mathrm{clip}}\right)$, $L_{\delta_{H}}\left(\cdot \right)$ is the Huber loss function, and ${\hat{R}}_{t}={\hat{A}}_{i,t}+V_{\theta_{\mathrm{old}}}\left(s_{t}\right)$ is the target return. The optimization objective of MAPPO is:

(30) $$L\left(\phi ,\theta \right)=L^{\mathrm{CLIP}}\left(\phi \right)-c_{1}L^{\mathrm{VF}}\left(\theta \right)+c_{2}H\left[\pi_{\phi }\right]$$

where $H[\pi_{\phi }]$ is the policy entropy, encouraging the agent to maintain exploration capabilities; $c_{1}$ and $c_{2}$ are balancing coefficients. Within each PPO training cycle, multiple rounds ($K_{\mathrm{epoch}}$ times) of mini-batch gradient updates are performed on the collected data, and gradient clipping alongside linear learning rate decay are utilized to ensure training stability. Specifically, the learning rate linearly decays according to:

(31) $$lr_{t}=lr_{0}\left(1-\frac{t}{T_{\max}}\right)$$

where $lr_{0}$ is the initial learning rate and $T_{\max}$ is the total number of training steps.

The complete training procedure of the algorithm is shown in Algorithm 1.
**Algorithm 1** Training Procedure Based on GR-MAPPO**Require:** Multi-agent environment E, communication graph construction rule, initialized shared policy network $\pi_{\phi }$ and value network $V_{\theta }$, learning rates $\alpha_{\phi },\alpha_{\theta }$, discount factor γ, GAE parameter λ, PPO clipping parameter $\epsilon_{\mathrm{clip}}$, PPO epochs $K_{\mathrm{epoch}}$, entropy value loss coefficient $c_{1}$, entropy coefficient $c_{2}$**Ensure:** Optimized shared policy network $\pi_{\phi^{*}}$ and value network $V_{\theta^{*}}$  1:Initialize: shared policy network $\pi_{\phi }$ (including GraphSAGE and GRU), shared value network $V_{\theta }$  2:**for** each episode **do**  3:      Reset environment, obtain initial observations $\left\{o_{i}^{0}\right\}$  4:      Initialize Actor GRU and Critic GRU hidden states $h_{i,0}^{\mathrm{gru}},h_{0}^{\mathrm{critic}}$  5:      Construct communication graph $G_{0}$ based on current defender positions  6:      **for** each time step **do**  7:            **for** each defender **do**  8:                   $f_{i}^{\mathrm{spatial}}\leftarrow \mathrm{GraphSAGE}\left(G_{t},\mathrm{LayerNorm}\left(o_{i}^{t}\right)\right)$  9:                   $f_{i}^{\mathrm{temporal}},h_{i,t}^{\mathrm{gru}}\leftarrow \mathrm{GRU}\left(f_{i}^{\mathrm{spatial}},h_{i,t-1}^{\mathrm{gru}}⊙{\mathrm{mask}}_{t}\right)$10:                  Sample action $a_{i}^{t}$ from $\pi_{\phi }\left(\cdot |o_{i},G_{t},h_{i,t-1}^{\mathrm{gru}}\right)$11:            **end for**12:            Compute $V_{\theta }\left(s_{t}\right)$ and update $h_{t}^{\mathrm{critic}}$13:            Execute joint actions, obtain rewards $\left\{r_{i,t}\right\}$ and new observations $\left\{o_{i}^{t+1}\right\}$14:            Update communication graph $G_{t+1}$15:            Store transition data into rollout buffer16:      **end for**17:      Compute GAE advantage function ${\hat{A}}_{t}$18:      **for** $k=1,\ldots ,K_{\mathrm{epoch}}$ **do**19:            Sample mini-batch data from buffer20:            Update policy network: $\phi \leftarrow \phi +\alpha_{\phi }\nabla_{\phi }L^{\mathrm{CLIP}}$21:            Update value network: $\theta \leftarrow \theta -\alpha_{\theta }\nabla_{\theta }L^{\mathrm{VF}}$22:      **end for**23:**end for**

**Remark** **1.** *The PPO implementation follows the MAPPO framework with shared policy parameters across defenders. The reported mini-batch size of 1 refers to the number of PPO mini-batches used for each rollout update, meaning that the collected rollout buffer from all 64 parallel environments is processed as a single batch without further subdivision. This setting follows common MAPPO implementations and was adopted to improve training stability under recurrent and graph-based policy structures.*

Although deriving a strict theoretical convergence guarantee for deep multi-agent reinforcement learning with recurrent and graph neural network structures remains challenging, the proposed GR-MAPPO framework inherits the stable optimization characteristics of PPO-based methods. Specifically, the clipped surrogate objective constrains excessive policy updates and improves training stability, while GAE reduces the variance of policy gradient estimation. In addition, gradient clipping and learning rate decay are adopted to further stabilize the optimization process. Similar convergence and stability discussions for PPO and MAPPO-based frameworks can be found in [21,28]. These mechanisms contribute to improving optimization stability during the training process.

## 4. Simulation Results and Analysis

### 4.1. Experiment Settings

The simulation environment is built upon the Multi-Agent Particle Environment (MPE) platform, adopting the two-dimensional bounded plane attack–defense scenario described in Section 2. A horizontal boundary line is set at y=2.0 above the field. The attackers’ starting line is set to $y_{\mathrm{spawn}}=-2.0$. Attackers are randomly generated from the lower area of the field (y∈[−3.0,−2.5]), and defenders are initially randomly generated in the middle area of the field (y∈[0,1.0]). The field width scales adaptively according to the number of defenders. Based on 3 defenders corresponding to a baseline width of $W_{base}=4.0$, when the number of defenders is *N*, the total field width is $W_{env}=N\times W_{base}/3$, and the *x*-axis range is set to $\left[-W_{env}/2,W_{env}/2\right]$. The default training scenario is set to 3 defenders against 3 attackers (3v3), with a corresponding field width of approximately 4.

The physical parameters of both the attacking and defending sides are shown in Table 1. The defending side adopts a second-order dynamics model, where the policy network outputs acceleration commands, inherently possessing an acceleration response delay. In contrast, the attacking side adopts a first-order kinematics model, where the policy network directly outputs velocity commands, allowing instantaneous changes in the motion state. Specifically, the defender dynamics are updated in discrete time as:

(32) $$v_{i}\left(t+1\right)=\mathrm{clip}\left(v_{i}\left(t\right)+\kappa a_{i}\left(t\right)\Delta t,\;-v_{\max},\;v_{\max}\right)$$

(33) $$p_{i}\left(t+1\right)=p_{i}\left(t\right)+v_{i}\left(t+1\right)\Delta t$$

where $a_{i}\left(t\right)$ is the acceleration command generated by the policy network, Δt=0.1 s is the simulation time step, and κ=1.0 is the acceleration scaling coefficient.

To compensate for the dynamic response disadvantage of the second-order system, the maximum velocity limit of the defending side (0.50 m/s) is set slightly higher than that of the attacking side (0.42 m/s), thereby ensuring the feasibility of the interception task. The detailed hyperparameter configuration of the training process is shown in Table 2. All experiments are based on the Python 3.8 environment, implemented using the PyTorch (2.3.0+cu12.1) framework and the Deep Graph Library (DGL).

**Remark** **2.** *The breach penalty coefficient −3.5 was selected empirically to balance the relative importance between successful interception rewards and defense failure penalties. Specifically, the breach penalty is designed to be larger than the dense distance-guided reward terms so that defenders prioritize boundary protection, while avoiding excessively large negative rewards that could destabilize policy optimization or induce overly conservative behaviors during training.*

Additionally, the specific hyperparameters defining the attackers’ multi-stage APF strategy are detailed in Table 3.

To systematically evaluate the independent contributions and synergistic effects of the GraphSAGE spatial aggregation module and the GRU temporal modeling module, four sets of ablation experiments were designed. All methods adopt the same hyperparameter configurations, reward functions, and training steps, differing only in the feature processing modules of the policy network. Each method is trained in data parallel across 64 environments and evaluated in scenarios under seeds 12, 24, and 36 to verify the stability and reproducibility of the performance. The experimental verification is divided into two stages: the first stage is the training scenario performance evaluation, where the convergence characteristics and final interception performance of the four methods are compared in the 3v3 training scenario, and the role of each module is analyzed through ablation experiments. The second stage is the zero-shot generalization evaluation, where the models trained in the 3v3 scenario are directly deployed to large-scale 10v10 and 20v20 scenarios.

### 4.2. Comparison Experiments

To verify the rationale of using MAPPO as the foundational improvement framework, this section conducts comparative experiments on three representative multi-agent reinforcement learning algorithms under the same 3v3 boundary defense environment. The three algorithms are: IPPO [29] based on the Independent Training Independent Execution paradigm, Qmix [30] based on the Centralized Training Decentralized Execution (CTDE) paradigm, and MAPPO [21] based on the CTDE paradigm. All three adopt the unified hyperparameter configurations listed in Table 2 and are trained under the same total training steps of 5 × 10^6^.

**Figure 4:**
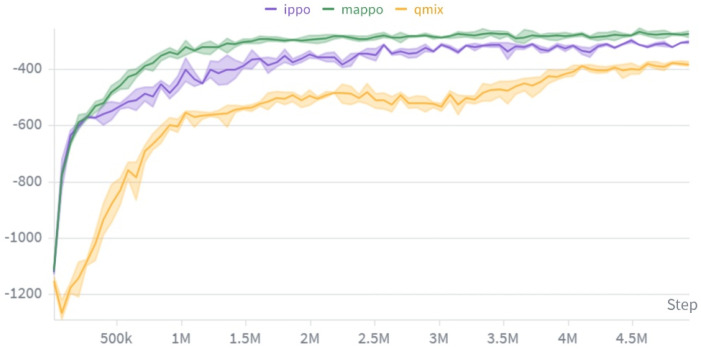
Rewards curves of MAPPO/IPPO/Qmix.

Figure 4 illustrates the mean cumulative reward curves of the three algorithms during the training process. The solid lines represent the mean values across 3 independent random seeds (seeds 12, 24, and 36), and the shaded regions denote the standard deviation. No curve smoothing is applied in order to present the raw training dynamics. The experimental results show that MAPPO outperforms the other two algorithms in both convergence speed and final performance.

Observing the average cumulative reward curves, all three algorithms enter a phase of rapid performance improvement after approximately $5\times 10^{5}$ steps. In the early stage of training, IPPO and MAPPO exhibit similar convergence speeds; however, starting from $1.5\times 10^{6}$ steps, MAPPO’s centralized value network gradually demonstrates its advantage, outperforming IPPO and converging to approximately −300 at $5\times 10^{6}$ steps, which is superior to IPPO’s approximate −320. In contrast, Qmix shows the weakest overall performance, with a slower convergence speed, eventually reaching only about −400, demonstrating a significant gap compared to MAPPO. Thus, in boundary defense tasks, MAPPO is more suitable for handling dynamic collaboration relying on global information, whereas Qmix has limited performance improvement constrained by the mixing network’s ability to express non-monotonic collaborative relationships.

MAPPO’s advantage in capture efficiency indicates that the global state information provided by the centralized value network helps agents achieve more effective interception of intruders in fewer time steps. The final average capture steps are shown in Table 4. Specifically, the “Average Capture Steps” metric is computed as the average capture time steps of successfully intercepted attackers. If no attackers are intercepted during an episode, the metric defaults to the maximum episode length ($T_{\max}$). Based on the above experimental results, this paper selects MAPPO as the foundational algorithm framework for subsequent improvements.

In summary, MAPPO exhibits the best baseline performance in the boundary defense task of this paper. Its CTDE framework and policy gradient optimization mechanism also provide a suitable extension foundation for the subsequent introduction of the GraphSAGE spatial aggregation module and the GRU temporal modeling module. Section 4.3 will progressively overlay each improved module onto MAPPO to analyze their independent roles and synergistic effects after combination.

### 4.3. Ablation Experiments

**Figure 5:**
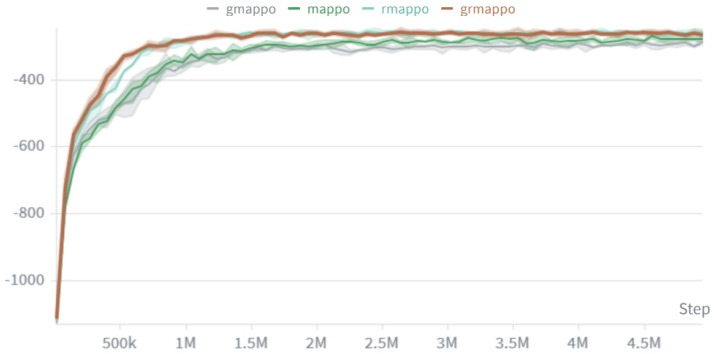
Reward functions in the 3v3 scenario.

Figure 5 presents the mean cumulative rewards of the four methods: MAPPO, G-MAPPO, R-MAPPO, and GR-MAPPO in the 3v3 scenario. Similar to the comparative experiments, the shaded bands indicate the standard deviation across different random seeds, and the curves are plotted without any smoothing. Overall, all four methods quickly achieve policy improvement in the early stages of training and gradually converge after approximately $1.5\times 10^{6}$ steps, with a generally stable training process. Combining the two metrics, R-MAPPO and GR-MAPPO perform the best, followed by MAPPO, while G-MAPPO is relatively weaker.

Specifically, R-MAPPO exhibits a faster rising speed and a higher convergence level in both the reward function and capture rate metrics, indicating that with the introduction of GRU, the policy network can effectively utilize historical observation information, thereby enhancing its modeling capability for the dynamic adversarial process. After about $1.5\times 10^{6}$ steps, the advantages of R-MAPPO begin to manifest stably, with its overall average cumulative reward being higher than MAPPO, and its capture rate approaching and remaining at a high level earlier.

In contrast, G-MAPPO shows only limited improvements compared to MAPPO, with insignificant gains on the reward curves. Although GR-MAPPO is roughly equivalent to R-MAPPO in final performance and shows slight fluctuating advantages in some training phases, it does not demonstrate a significant trend of surpassing R-MAPPO overall. This indicates that in the small-scale 3v3 scenario, structural modeling based on GraphSAGE contributes relatively little to performance improvement; by contrast, temporal information modeling is more critical for policy optimization.

Table 5 summarizes the final evaluation results of the four methods in the 3v3 scenario. After training is completed, each method is tested for 200 episodes using its final checkpoint, reporting the Mean Interception Rate (MIR) and Mean Average Reward (MAR).

Comparing the evaluation results of the above four algorithms, it can be concluded that the GRU temporal modeling module brings the most significant performance gain in the 3v3 scenario. Taking the comparison between MAPPO and R-MAPPO as an example, after introducing the GRU, the average capture rate increases from 87.05% to 97.84%, a growth of approximately 10 percentage points. This suggests that temporal memory capacity is a key factor affecting defense performance when facing attackers with multi-stage deceptive maneuvers. Since attackers exhibit distinctly different motion patterns during the probing approach stage and the sprinting stage, a single-frame observation is often insufficient to accurately identify their behavioral stages. The GRU, however, can encode historical motion information through its hidden state, thereby helping defenders judge the attacker’s behavior more accurately.

The effect of the GraphSAGE spatial aggregation module shows a dual nature in the 3v3 training scenario. Introducing GraphSAGE yields limited improvements in the capture rate; comparing R-MAPPO with GR-MAPPO, introducing GraphSAGE actually causes the capture rate to drop from 97.84% to 95.71%, a performance regression of about 2.1 percentage points. In the 3v3 small-scale scenario, the GraphSAGE module does not bring obvious performance gains. Considering the environmental parameters, the number of effective communication neighbors for defenders at this scale is small, which restricts the scope of the spatial aggregation mechanism, making its advantages difficult to manifest. With a field width of $W_{env}=4.0$ and a communication range of $R_{com}=0.88$, the average distance between the 3 defenders is approximately 1.33 m, which exceeds the communication range. In most time steps, each defender has only 0 to 1 communication neighbor, and the extra network layers conversely increase optimization difficulty. Furthermore, as an inductive graph neural network, the design goal of GraphSAGE is to learn a generalized neighbor aggregation function. This capability has not been fully utilized in fixed-scale training scenarios, and its true value will be demonstrated in the generalization experiments in Section 4.4.

### 4.4. Generalization Performance

Section 4.3 indicates that GraphSAGE provides limited contributions in the 3v3 scenario, but the design goal of this module is not to improve small-scale scenario performance, but to enhance the model’s cross-scale generalization capability. To verify this, this section conducts zero-shot transfer experiments.

**Figure 6:**
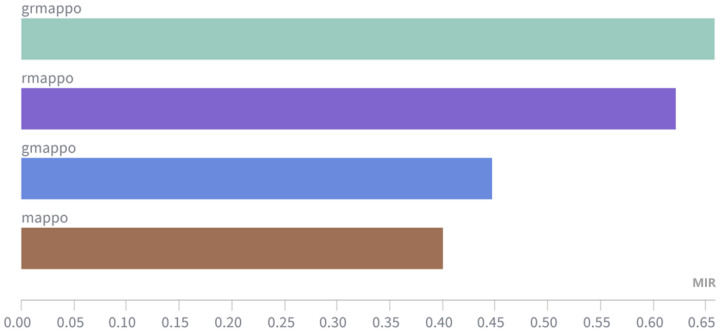
Evaluation performance in the 10v10 generalization scenario.

The final models of the four methods (MAPPO, G-MAPPO, R-MAPPO, and GR-MAPPO) trained in the 3v3 scenario are directly deployed for testing in 10v10 and 20v20 scale scenarios without any parameter adjustment. The feasibility of this experiment is established on the two design foundations described in Section 3: the scale-invariance of observation dimensions and the consistency of environmental density. To ensure the fairness of the generalization evaluation, all methods are trained in the 3v3 scenario under identical training settings and directly tested in larger-scale scenarios. Figure 6 shows the evaluation results of the four methods in the 10v10 generalization scenario. Unlike the situation in the 3v3 scenario where the performance of all methods was close, the four methods exhibit performance stratification in the 10v10 scenario.

The performance gap between GR-MAPPO and R-MAPPO widens significantly in the 10v10 scenario, contrasting with their essentially equivalent performance in the 3v3 scenario, which directly reflects the role of the GraphSAGE module in scale generalization. In the 10v10 scenario, the field width expands to $W_{env}\approx 13.3$, and the communication range correspondingly extends to $R_{com}\approx 2.93$. The communication neighbors of each defender increase from no more than 2 in the 3v3 scenario to about 4, enabling GraphSAGE to aggregate more friendly information. Thus, GR-MAPPO demonstrates a more pronounced performance advantage over R-MAPPO.

The performance of R-MAPPO is superior to MAPPO and G-MAPPO in the 10v10 scenario. This shows that when facing attackers with deceptive maneuvers, the temporal modeling capability is still an indispensable foundational capability, and GraphSAGE’s spatial collaboration advantage is still not fully utilized at the current scale.

**Figure 7:**
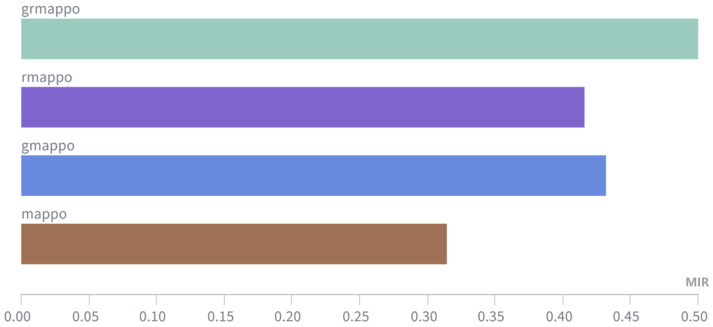
Evaluation performance in the 20v20 generalization scenario.

As shown in Figure 7, the advantage of GR-MAPPO is even more prominent in the 20v20 scenario: the average capture rate of GR-MAPPO is approximately 50%, maintaining the lead among the four methods. R-MAPPO reaches about 41.6%, further widening the gap with GR-MAPPO. Notably, at this point, the performance of G-MAPPO surpasses R-MAPPO, suggesting that the spatial information aggregation capability among agents plays a stronger role than the temporal dependency modeling capability in this scenario. The capture rate of MAPPO drops to approximately 31.4%, suffering the most severe performance decay.

As the number of agents increases from 3 to 20, the performance gap between GR-MAPPO and the other methods continues to expand with scale. Table 6 quantitatively summarizes this trend.

To further investigate whether the observed generalization performance is mainly caused by enlarged sensing and communication ranges in larger-scale scenarios, we additionally conducted controlled experiments under a fixed-radius setting. Specifically, during the 10v10 and 20v20 testing phases, both the defender sensing range $R_{det}$ and communication range $R_{com}$ are fixed to the same values used in the 3v3 training scenario, instead of scaling proportionally with the environment size. The corresponding results are summarized in Table 6.

To quantitatively evaluate the cross-scale generalization capability of the models, the ‘Performance Decay’ metric in Table 6 is defined as:

(34) $$\Delta_{\mathrm{MIR}}={\mathrm{MIR}}_{3v3}-{\mathrm{MIR}}_{20v20}$$

i.e., the difference in capture rates between the 3v3 training scenario and the 20v20 generalization scenario, which is used to measure the performance retention capability of each method during scale extrapolation. A smaller value indicates stronger generalization.

According to Table 6, under the scaled-radius setting, the performance gap between R-MAPPO and GR-MAPPO monotonically increases with scale; the two are roughly equivalent at 3v3, the gap is about 3.6% at 10v10, and widens to about 9.7% at 20v20. This trend is directly attributed to the permutation invariance of GraphSAGE’s mean aggregation. This characteristic allows the aggregation function learned at 3v3 to be directly applied to large-scale scenarios with more neighbors, while the inductive learning paradigm enables the model to generalize to graph topologies unseen during training.

As shown in Table 6, although all methods exhibit lower capture rates under the fixed-radius setting due to restricted information access and increased coordination difficulty, GR-MAPPO still consistently achieves the best performance among all baselines. In particular, under the fixed-radius setting, GR-MAPPO achieves 55.40% and 43.80% capture rates in the 10v10 and 20v20 scenarios, respectively, outperforming R-MAPPO by an absolute margin of 4.2% and 11.1%.

These results indicate that the superior cross-scale generalization capability of GR-MAPPO does not merely rely on enlarged sensing or communication ranges. Instead, the GraphSAGE-based relational aggregation mechanism itself contributes substantially to transferable coordination under limited local information, enabling the model to maintain effective multi-agent collaboration even when the perceptual and communication capabilities remain unchanged across scales.

Synthesizing the experimental results across the three scales, it can be concluded that GR-MAPPO maintains the highest capture rate in all generalization test scales, and its relative advantage expands with scale. The GRU module provides a stable performance gain across all scales, whereas the contribution of the GraphSAGE module significantly increases as the scale grows.

## 5. Conclusions

Addressing the multi-agent boundary defense problem under local communication constraints and dynamically changing swarm scales, this paper proposes a cooperative defense strategy integrating GraphSAGE spatial aggregation and GRU temporal modeling. The method utilizes MAPPO as the foundational training framework and designs a composite reward function that balances dense guidance with sparse event feedback to drive policy optimization. Within this framework, GraphSAGE is employed to dynamically construct a communication graph among defenders and aggregate friendly collaborative features, while the GRU encodes historical observation sequences to capture the attackers’ phased behavioral shifts. Ablation experiments and cross-scale generalization experiments indicate that the two modules play distinct yet complementary roles within the model. The temporal modeling capability provided by the GRU is an important factor in improving defense performance across all test scales, particularly when dealing with attackers exhibiting deceptive maneuvering behaviors. GraphSAGE’s spatial aggregation capacity offers limited contributions in small-scale scenarios, but its importance rises rapidly as the scale increases. In the 20v20 scenario, the standalone GraphSAGE model (G-MAPPO) already outperforms R-MAPPO, which only includes GRU, reflecting that the constraint of ‘cooperative division of labor’ on performance gradually surpasses that of ‘intent inference’ in large-scale scenarios. GR-MAPPO, which incorporates both modules, achieves the optimal performance across all test scales, and its advantage expands with increasing scale, validating the effectiveness of the proposed network architecture.

Nevertheless, several directions remain for future research. First, the current work mainly considers cooperative defense in simplified simulation environments. Extending the framework to more complex scenarios involving dynamic obstacles, communication delays, or adversarial communication interference would further improve its practical applicability. Second, although the proposed method demonstrates favorable performance retention under cross-scale transfer, training efficiency and scalability under ultra-large-scale swarm settings still deserve further investigation. Curriculum learning or hierarchical training strategies may help improve learning stability and accelerate convergence in more challenging tasks. In addition, future work may explore more robust communication and coordination mechanisms to enhance adaptability under highly dynamic and uncertain environments.

## Figures and Tables

**Table 1:** Physical parameters of agents.

Parameter	Description	Attacker	Defender
$r_{agent}$	Agent radius (m)	0.04	0.04
$v_{max}$	Max velocity (m/s)	0.42	0.50
$R_{det}$	Sensing range (m)	0.3	$0.4W_{env}$
$R_{com}$	Communication range (m)	—	$0.22W_{env}$
$R_{cap}$	Capture radius (m)	—	0.2
$k_{a}$	Max perceived attackers	—	min(M,3)

**Table 2:** Training parameter.

Parameter Type	Parameter Name	Value
Environment	Max interaction steps	200
	Total training steps	5,000,000
Learning	Actor learning rate	7 × $10^{-4}$
	Critic learning rate	7 × $10^{-4}$
	GAE Lambda λ	0.95
	Discount factor γ	0.99
	PPO clip parameter $\epsilon_{\mathrm{clip}}$	0.2
	Value loss coefficient $c_{1}$	1.0
	Entropy coefficient $c_{2}$	0.01
	PPO update epochs	10
	Mini-batch size	1
	Optimizer	Adam
	Value loss function	Huber Loss
	Network initialization	Orthogonal initialization
Reward	Team capture reward $r_{i,team}$	+2.0
	Individual capture reward $r_{i,individual}$	+8.0
	Breach penalty $\lambda_{breach}$	−3.5
Network	Hidden layer dimension $d_{h}$	64
	MLP layers	2
	GRU layers	1
	Activation function	Tanh
	GraphSAGE layers	2
	GraphSAGE aggregation	Mean

**Table 3:** Hyperparameters of the attackers’ multi-stage APF strategy.

Parameter Type	Parameter	Value
Stage Thresholds	Stage 1 transition $\mu_{th1}$	0.4
	Stage 2 transition $\mu_{th2}$	0.75
	Sprint bypass progress $\mu_{\mathrm{sprint}}$	0.3
	Sprint bypass threat $\lambda_{\mathrm{threat}}^{\mathrm{th}}$	0.8
Base Force Gains	Attractive gain ζ	0.3
	Repulsive gain $\eta_{\mathrm{rep}}$	0.1
	Repulsive sensing radius $d_{0}$ (m)	0.3
Ally Interaction	Ally attraction gain $\eta_{\mathrm{att}}$	0.2
	Ally repulsion gain $\eta_{\text{rep\_ally}}$	0.3
	Minimum distance $d_{\mathrm{ally}}^{\min}$ (m)	0.3
	Maximum distance $d_{\mathrm{ally}}^{\max}$ (m)	1.5
Stage-dependent Oscillation	Stage 1 ($\omega_{1}$/A(1))	0.5/(0.6, 0.8)
	Stage 2 ($\omega_{2}$/A(2))	0.8/(0.4, 0.6)
	Stage 3 ($\omega_{3}$/A(3))	1.5/(0.3, 0.4)
Stage Modulators	Stage 1 ($\alpha_{1},\beta_{1},\gamma_{1},\delta_{1}$)	0.3, 2.0, 2.0, 0.5
	Stage 2 ($\alpha_{2},\beta_{2},\gamma_{2},\delta_{2}$)	0.8, 1.5, 1.5, 2.0
	Stage 3 ($\alpha_{3},\beta_{3},\gamma_{3},\delta_{3}$)	1.5, 0.8, 1.0, 1.0
Noise Scale	Stage 1 ($\sigma_{o,1}$)	0.15
	Stage 2 ($\sigma_{o,2}$)	0.08
	Stage 3 ($\sigma_{o,3}$)	0.05

**Table 4:** Summary of final evaluation performance for the three algorithms.

Algorithm	Training Paradigm	Average Cumulative Reward	Average Capture Steps	MIR
IPPO	Independent Training, Independent Execution	−298.87	82.92	0.9096
Qmix	CTDE (Value Decomposition)	−390.52	85.81	0.8271
MAPPO	CTDE (Policy Gradient)	−286.06	78.97	0.9334

**Table 5:** Evaluation performance in the 3v3 scenario.

Method	GraphSAGE	GRU	Mean Interception Rate MIR (%)	Mean Cumulative Reward MAR
MAPPO	✕	✕	87.05% ± 5.24%	−299.69 ± 17.42
R-MAPPO	✕	✓	97.84% ± 1.71%	−259.72 ± 10.91
G-MAPPO	✓	✕	87.53% ± 6.69%	−289.22 ± 18.42
Ours	✓	✓	95.71% ± 3.15%	−272.04 ± 13.65

Note: ✕ means not used, ✓ means used.

**Table 6:** Comparison of average capture rates (%) under scaled-radius and fixed-radius settings across different scales.

Method	3v3 Training	Scaled $R_{\det},R_{\mathrm{com}}$			Fixed $R_{\det},R_{\mathrm{com}}$		
		10v10	20v20	Decay	10v10	20v20	Decay
MAPPO	87.05 ± 5.24	40.92 ± 7.76	31.40 ± 11.79	55.65	34.50 ± 8.25	23.80 ± 9.51	63.25
R-MAPPO	**97.84 ± 1.71**	62.17 ± 4.53	41.64 ± 8.36	56.21	51.20 ± 6.37	32.70 ± 8.19	65.14
G-MAPPO	87.53 ± 6.69	44.68 ± 7.11	42.20 ± 7.96	45.33	38.60 ± 7.43	34.20 ± 7.90	53.33
GR-MAPPO	95.71 ± 3.15	**65.83 ± 5.24**	**51.37 ± 7.12**	**44.34**	**55.40 ± 5.82**	**43.80 ± 7.33**	**51.91**

Note: The best metrics are highlighted in bold.

## Data Availability

The data and code that support the findings of this study are available from the corresponding author upon reasonable request. The code is not publicly available due to ongoing research and development.
